# Locoregional Treatment in De Novo Bone-Only Metastatic Breast Cancer: Prospective, Multi-Institutional Real-World Data, BOMETIN, Protocol MF14-1a

**DOI:** 10.3390/curroncol32100556

**Published:** 2025-10-03

**Authors:** Atilla Soran, Berk Göktepe, Berkay Demirors, Ozgur Aytac, Serdar Ozbas, Lutfi Dogan, Didem Can Trablus, Jamila Al-Azhri, Kazım Senol, Shruti Zaveri, Salyna Meas, Umut Demirci, Hasan Karanlik, Aykut Soyder, Ahmet Dag, Ahmet Bilici, Mutlu Dogan, Mehmet Ali Nahit Sendur, Hande Koksal, Mehmet Ali Gulcelik, Neslihan Cabioglu, Levent Yeniay, Zafer Utkan, Nuri Karadurmus, Gul Daglar, Turgay Simsek, Birol Yildiz, Cihan Uras, Mustafa Tukenmez, Cihangir Ozaslan, Niyazi Karaman, Arda Isik, Efe Sezgin, Vahit Ozmen, Anthony Lucci

**Affiliations:** 1 Department of Surgery, University of Pittsburgh, Pittsburgh, PA 15213, USA; demirorsb2@upmc.edu; 2 Department of General Surgery, Faculty of Medicine, Ege University, Izmir 35100, Türkiye or berk.goktepe@ege.edu.tr (B.G.); levent.yeniay@ege.edu.tr (L.Y.); 3 Department of General Surgery, Faculty of Medicine, Baskent University, Adana 01250, Türkiye; oaytac@gmail.com; 4 Breast Surgery, Private Practice, Ankara 06680, Türkiye; sozbas@yahoo.com; 5 Department of Surgical Oncology, Dr Abdurrahman Yurtaslan Ankara Oncology Training and Research Hospital, University of Health Sciences, Ankara 06200, Türkiye or lutfi.dogan@sbu.edu.tr (L.D.); cihangir.ozaslan@sbu.edu.tr (C.O.); 6 Department of General Surgery, Medical Park Goztepe, Faculty of Medicine, Bahçeşehir University, Istanbul 34732, Türkiye; didemcan73@gmail.com; 7 Department of Cancer Prevention and Control, Roswell Park Cancer Institute, Buffalo, NY 14263, USA; drjamila@alazhri.com; 8 Department of General Surgery, Faculty of Medicine, Uludag University, Bursa 16059, Türkiye; kazimsenol@gmail.com; 9 Department of Surgery, The University of Texas Southwestern Medical Center, Dallas, TX 75390, USA; shruti.zaveri@utsouthwestern.edu; 10 Department of Breast Surgical Oncology, The University of Texas MD Anderson Cancer Center, 1400 Pressler Street, FCT 7.6000, Unit 1484, Houston, TX 77030, USA; smeas@mdanderson.org (S.M.); alucci@mdanderson.org (A.L.); 11 Department of Medical Oncology, Memorial Ankara Hospital, Ankara 06520, Türkiye; umut.demirci@memorial.com.tr; 12 Department of Surgical Oncology, Istanbul University Institute of Oncology, Istanbul 34093, Türkiye; hasankaranlik@yahoo.com; 13 Department of General Surgery, Faculty of Medicine, Acıbadem Mehmet Ali Aydınlar University, Istanbul 34662, Türkiye or aykut.soyder@acibadem.com.tr (A.S.); cihan.uras@acibadem.com (C.U.); 14 Department of General Surgery, Faculty of Medicine, Mersin University, Mersin 33343, Türkiye; dahmetdag@yahoo.com; 15 Department of Medical Oncology, Faculty of Medicine, Medipol University, Istanbul 34214, Türkiye; abilici@medipol.edu.tr; 16 Department of Medical Oncology, Dr Abdurrahman Yurtaslan Ankara Oncology Training & Research Hospital, University of Health Sciences, Ankara 06200, Türkiye; mutlu.dogan@sbu.edu.tr; 17 Department of Medical Oncology, Faculty of Medicine, Ankara Yıldırım Beyazıt University, Ankara 06690, Türkiye; masendur@yahoo.com.tr; 18 Department of General Surgery, Faculty of Medicine, Selçuk University, Konya 42130, Türkiye; drhandeniz@yahoo.com; 19 Department of Surgical Oncology, Health Sciences University, Faculty of Medicine, Ankara Gülhane Research and Training Hospital, Ankara 06010, Türkiye; mehmetali.gulcelik@sbu.edu.tr; 20 Department of General Surgery, Istanbul Faculty of Medicine, Istanbul University, Istanbul 34093, Türkiye or neslihan.cabioglu@istanbul.edu.tr (N.C.); mustafa.tukenmez@istanbul.edu.tr (M.T.); 21 Department of General Surgery, Faculty of Medicine, Kocaeli University, İzmit 41001, Türkiye or zafer.utkan@kocaeli.edu.tr (Z.U.); turgay.simsek@kocaeli.edu.tr (T.S.); 22 Department of Medical Oncology, Health Sciences University, Faculty of Medicine, Ankara Gülhane Research and Training Hospital, Ankara 06010, Türkiye; ns23@aub.edu; 23 Private, Breast and Endocrine Surgeon, Ankara 06520, Türkiye; drguldaglar@gmail.com; 24 Department of Medical Oncology Dr Rıdvan Ege Hospital, Ufuk University, Ankara 06510, Türkiye; bfyildiz@gmail.com; 25 Department of General Surgery, Dr Abdurrahman Yurtaslan Ankara Oncology Training and Research Hospital, University of Health Sciences, Ankara 06200, Türkiye; niyazikaraman@hotmail.com; 26 Department of Surgery, Faculty of Medicine, Istanbul Medeniyet University, Istanbul 34720, Türkiye; kararda@yahoo.com; 27 Department of Food Engineering, Izmir Institute of Technology, Izmir 35433, Türkiye; efesezgin@iyte.edu.tr; 28 Breast Center, Istanbul Florence Nightingale Hospital, Istanbul 34381, Türkiye; vahitozmen@yahoo.com.tr

**Keywords:** metastatic breast cancer, bone, primary tumor surgery

## Abstract

*Introduction*: The impact of locoregional treatment (LRT) on survival in de novo bone-only metastatic breast cancer (dnBOMBC) is controversial. This study aims to assess the effect of LRT on survival, utilizing international, prospectively acquired data in this cohort of patients. *Materials and Methods*: Patients with dnBOMBC were divided into two groups: those receiving systemic therapy only (ST) and those undergoing LRT. Further, patients who received LRT were divided into two subgroups: those who received ST after LRT (LRT+ST group) and those who received ST prior to LRT (ST+LRT group). Factors associated with disease progression, including solitary or multiple bone metastases, were analyzed. *Results*: There was a total of 744 patients with dnBOMBC treated at each of the participating institutions between 2014 and 2022, with 372 (50%) participants in each arm. Median follow-up was 48 months (32–66, 25–75%). Patients in the LRT group were significantly younger than the ST group [50 (42, 60) vs. 55 (44, 66), *p* = 0.0001]. There were no significant differences in grade, HER2 status, triple-negative status, receipt of hormonal therapy, or intervention to metastatic sites. During follow-up, 58% (*n* = 217) of patients in the ST group and 32% (*n* = 120) of patients in the LRT group died (*p* < 0.001). Local progression was observed in 20% of the patients in the ST group, whereas 9% progressed in the LRT group (*p* = 0.0001). Systemic progression occurred more in the ST group; 66% (*n* = 244) compared to 41% (*n* = 152) of patients in the LRT group (*p* < 0.001). The hazard of death was 64% lower in the LRT group than in the ST group (HR: 0.36, 95% CI: 0.29–0.45, *p* < 0.0001). The burden of metastatic disease differed significantly between the two groups, with a higher rate of solitary bone metastases in the LRT group compared to the ST group (50% vs. 24%, *p* < 0.001). However, the LRT group had better overall survival (OS) for both solitary (HR: 0.38, 95% Cl: 0.26–0.55) and multiple (HR: 0.38, 95% Cl: 0.29–0.51) bone metastasis patients. Within the LRT group, survival rates were similar whether the breast surgery was performed before or after ST. Multivariate Cox analysis showed that LRT and ER/PR positivity significantly decrease the hazard of death (*p* < 0.05). *Conclusions*: Analysis of this large multi-institutional patient cohort provides further evidence that LRT is associated with longer OS and lower locoregional recurrence rates in patients with dnBOMBC. In breast cancer patients with bone-only metastases at presentation, the decision for LRT should be made through a multidisciplinary approach with consideration of surgical therapy at the primary tumor.

## Simple Summary

De novo metastatic breast cancer is characterized by the presence of distant metastases at initial diagnosis and accounts for about 6–10% of all new breast cancers. While systemic therapy remains the standard approach, this is a very heterogeneous disease, including diverse subgroups with varying outcomes. Our study presents the largest set of international, prospectively collected data, highlighting a favorable subgroup in which local and regional treatments were associated with improved overall survival.

## 1. Introduction

Breast cancer (BC) is the most common cancer diagnosed among women and the second leading cancer-related mortality reason worldwide [1]. Approximately 6–10% of newly diagnosed BCs in the United States are categorized as de novo metastatic breast cancer (dnMBC), characterized by the presence of distant metastases at initial diagnosis [2]. The median survival time of patients with metastatic BC was 2 to 3 years, but this has significantly improved in a selected group of patients; up to 13% of dnMBC patients can survive beyond 10 years [3,4]. With the advent of targeted therapies, cyclin-dependent kinase (CDK) 4/6 inhibitors, human epidermal growth factor receptor 2 (HER2)-directed agents, and multidisciplinary management, survival outcomes have nearly doubled, particularly in patients with bone-only metastases, hormone receptor (HR)-positive, and/or HER2-positive tumors.

Systemic therapies (ST) remain the mainstay of dnMBC treatment, especially in controlling disease progression and prolonging survival. However, in patients with favorable biology and indolent disease, the prolonged ST treatment duration can result in cumulative toxicity, resistance, and locoregional progression (LP), which reduces the quality of life [5]. The role of locoregional treatment (LRT), which includes surgical resection and/or radiotherapy (RT) directed at the primary breast tumor, has gained increasing importance as a potential strategy to improve outcomes in select dnMBC patients [6]. International current practice guidelines have different recommendations. The National Comprehensive Cancer Network (NCCN) states that LRT in de novo stage IV disease may be considered only after a favorable response to ST, highlighting that the survival benefit of upfront surgery remains unclear [7]. Meanwhile, the European Society for Medical Oncology (ESMO) suggests that the true value of LRT is still unknown but supports its selective use in carefully chosen patients, particularly those with limited disease burden and good performance status [8].

LRT improves local disease control and reduces the progression of the breast tumor. However, its impact on overall survival (OS) remains controversial. Multiple retrospective studies have provided mixed results; while some show no significant benefit, others reported improved survival in patients undergoing LRT, especially those with bone-only disease and low metastatic burden [9,10]. Meta-analyses suggest that certain subgroups, such as HR-positive and bone-only metastases, may have a survival benefit [11]. While randomized controlled trials (RCTs) are controversial regarding OS benefit from LRT, regardless of surgical timing, negative studies are often criticized due to limitations such as small sample sizes, treatment imbalances, low rates of HER2-targeted therapy, and selection bias [12,13,14]. However, it is important to note that LRT is associated with better locoregional control and longer progression-free survival compared to ST alone in these RCTs.

Bone is the most frequent site of distant metastasis in BC, with up to 75% of distant metastasis being bone metastasis, and among patients presenting with dnMBC, approximately 30–40% have bone-only metastases at diagnosis [15]. This subgroup often demonstrates a more indolent clinical course and favorable prognosis, particularly when associated with HR-positive or HER2-positive disease. Given the relatively better prognosis and unique disease dynamics of de novo bone-only metastatic breast cancer (dnBOMBC), this population presents a compelling opportunity to explore treatment strategies beyond ST alone. In our previous prospective multicenter BOMET study (MF14-01), we demonstrated that LRT was associated with improved OS and reduced LP compared to ST alone [2]. The current BOMETIN (MF14-1a) study is a direct expansion of that registry, in which additional patients were recruited from multiple international centers, and follow-up was extended, thereby substantially increasing the overall sample size. By analyzing real-world data, this study aims to clarify whether LRT confers a survival benefit in this uniquely prognostic and clinically relevant patient population.

## 2. Materials and Methods

The BOMETIN (MF14-1a) registry represents an updated and expanded version of the earlier MF14-01 study. All patients previously enrolled in MF14-01 were retained in the database, and additional patients meeting the same eligibility criteria were prospectively recruited from multiple centers. The present analysis, therefore, reflects a larger and more representative cohort, with increased statistical power compared to MF14-01, while maintaining methodological continuity.

Analyses were conducted considering two groups: those receiving ST only (ST group) and those receiving LRT (LRT group), even though the treating physicians determined the treatment sequence. The patients who received LRT were subsequently divided into two groups: ST before LRT (ST+LRT group) and ST after LRT (LRT+ST group).

LRT was defined as treatment for the primary breast/axilla and consisted of surgery with or without adjuvant RT. Surgical procedures included breast-conserving surgery (BCS) or mastectomy, with sentinel lymph node biopsy and axillary lymph node dissection per institutional practice. Margin status and RT administration (yes/no) were noted. Because this was a multi-institutional registry, dose, fractionation, boost use, and nodal-field specifications were not uniformly captured. Procedures directed at metastatic sites were analyzed separately and not included as LRT.

The diagnosis was made utilizing bone scintigraphy, magnetic resonance imaging (MRI), and Positron Emission Tomography–Computerized Tomography (PET/CT). Although it was not required, bone metastases were preferably confirmed by bone biopsy. If a bone biopsy was not performed, at least two imaging modalities were required for single bone metastases. Bone biopsies to confirm the metastases were performed on only 15% of the patients (115/744). The absence of any metastases other than bone metastases was confirmed by PET/CT, computed tomography (CT), MRI, ultrasonography, or chest X-rays. While the treating physicians retained discretion over all treatment options and choices for primary tumors and metastases, patients with HER2-positive tumors received anti-HER2 therapy, and patients with HR-positive tumors received endocrine therapy. Until they died or the statistical analysis was completed, patients were monitored every three to six months. OS was defined as the time from diagnosis to death; LP was defined as the progression or recurrence in the breast/chest wall or regional nodes; and systemic progression (SP) was defined as new or progressive distant disease, including bone or visceral organ metastases, recorded and analyzed.

### Statistical Analysis

**Figure 1:**
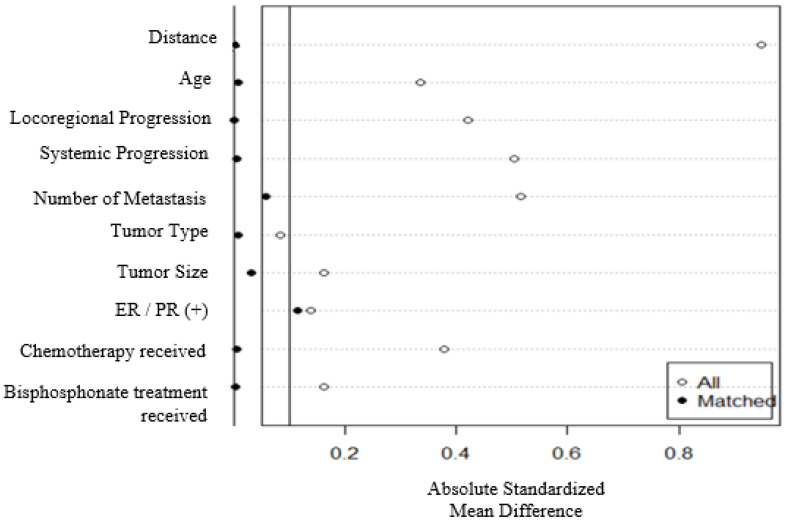
Standardized mean differences of confounding covariates before and after propensity score matching.

LRT and ST groups were analyzed using t-tests, Chi-square tests, the Shapiro–Wilk test, and the Kruskal–Wallis test were appropriate. Kaplan–Meier log-rank tests are used for survival analyses. Univariate and multivariate Cox proportional hazard models were applied to assess OS in relation to baseline demographic, clinical, tumor, and metastatic variables. Hazard ratios (HRs) with 95% confidence intervals (CI) were estimated. The proportional hazards assumption was evaluated for each group. For all comparisons and analyses, the proportional hazards assumption was met (*p* > 0.20). To account for the effect of confounding covariates on survival, in addition to multivariable Cox models, ST and LRT groups were matched based on significantly different clinicopathological factors, including age, tumor size, number of metastases, treatment type, LP, and SP, using a propensity score matching method. Full matching gave the best matching and adequate balance (Figure 1). Balance was assessed using standardized mean differences (target |SMD| < 0.10). The propensity score was estimated using probit regression of the treatment on the covariates. Full matching [16,17] used all treated and all control units, so no units were discarded by the matching. Kaplan–Meier methods were applied for survival curves for the ST and LRT cohorts within the matched dataset. Survival differences between groups were assessed using stratified log-rank tests, with stratification based on matched sets [18]. Weighted Cox regression models, including subclasses as a cluster [19], were used to estimate marginal HR and 95% CI. *p*-values < 0.05 were considered statistically significant. All analyses were conducted in R version 3.6.1 (R Foundation for Statistical Computing, Vienna, Austria, https://www.r-project.org) software packages (accessed on 5 April 2025). ‘MatchIt’ package (https://cran.rproject.org/web/packages/MatchIt/MatchIt.pdf; Version 4.2.0, 26 May 2021) (accessed on 5 April 2025) was used to implement propensity score matching [16,17,18,19].

## 3. Results

A total of 744 patients with dnBOMBC were treated across the participating institutions between 2014 and 2022. Of these, 372 participants (50%) received ST alone, and 372 (50%) underwent LRT. Within the LRT cohort, 151 patients (40.6%) received ST before primary breast surgery (ST+LRT group), while 221 patients (59.4%) underwent breast surgery prior to ST (LRT+ST group) (Table 1 and Table 2). No patient received RT alone; LRT always included surgery. All BCS cases received adjuvant RT per institutional standards. All surgical margins were negative. Median follow-up was 48 months (IQR 25–75%: 32–66), and it was 39 months and 58 months in the ST and LRT groups, respectively (*p* < 0.001). Patients in the LRT group were younger than the ST group (median age 50 vs. 55 years, *p* = 0.0001) with no body mass index (BMI) differences (median 27 kg/m^2^ vs. 28 kg/m^2^, *p* = 0.39). Solitary bone metastasis was significantly more common in the LRT group (50% vs. 24%, *p* < 0.001). The ST group had more multiple metastases (76% vs. 50%), and a higher number of T1 and T2 stage tumors (*p* = 0.009) (Table 1).

Invasive ductal carcinoma (IDC) predominated in both groups but was more common in the LRT cohort (84% vs. 77%, *p* = 0.0005).

There were no significant differences in HER-2-positive (28% vs. 25%, *p* = 0.36) and triple-negative BCs (7% vs. 5%, *p* = 0.28). Estrogen/Progesterone receptor (ER/PR)-positive tumor was lower in the LRT group (84% vs. 89%, *p* = 0.04), although hormonal therapy was administered equally (85% in both groups, *p* = 0.99). Chemotherapy administration was higher in the LRT group (95% vs. 87%, *p* = 0.0005), and bisphosphonate treatment was lower in the LRT group (62% vs. 70%, *p* = 0.02). Ovarian suppression therapy use was similar (24% in LRT vs. 19% in ST; *p* = 0.13). Intervention to metastatic sites was comparable between groups (53% LRT vs. 51% ST; *p* = 0.46).

In subgroup analysis, solitary metastasis was more common in the LRT+ST group (57%) than in the ST+LRT group (40%) (*p* < 0.001). Chemotherapy use was slightly higher in the ST+LRT group (98%) compared to LRT+ST (93%) and ST group (87%) (*p* = 0.0002). Bisphosphonate treatment was significantly less frequent in ST+LRT (54%) compared to LRT+ST (67%) and ST (70%) (*p* = 0.003). Ovarian suppression therapy rates were similar between subgroups (Table 2).

During follow-up, 58% (*n* = 217) of patients in the ST group and 32% (*n* = 120) of patients in the LRT group died (*p* < 0.001). SP occurred more frequently in the ST group, affecting 66% (*n* = 244) of patients, compared to 41% (*n* = 152) in the LRT group, and it was 39% (*n* = 87) in LRT+ST and 43% (*n* = 65) in ST+LRT (*p* < 0.001). LP was observed in 20% (*n* = 76) of patients in the ST group, compared to 9% (*n* = 32) in the LRT group (*p* = 0.0001). When stratifying within the LRT cohort, mortality was lower in both LRT+ST (34%) and ST+LRT (29%) groups compared to the ST group (58%) (*p* < 0.001).

**Figure 2:**
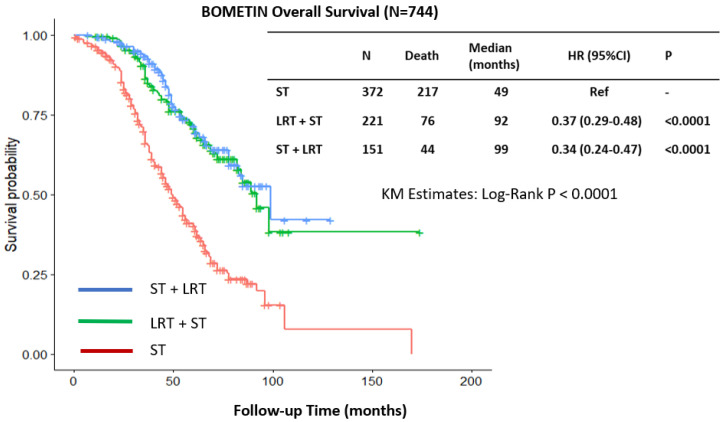
Overall survival regarding systemic therapy (ST) and locoregional therapy (LRT); subgroups defined as LRT+ST and ST+LRT groups.

In the overall cohort (*n* = 744), median OS was 49 months for the ST group, and 92 months (HR, 0.37; 95% CI, 0.29–0.48; *p* < 0.0001) for the ST+LRT and it was 99 months (HR, 0.34; 95% CI, 0.24–0.47; *p* < 0.0001) for the LRT+ST (Figure 2). Overall, the LRT group was associated with significantly improved OS compared to the ST group (HR, 0.36; 95% CI, 0.29–0.45; *p* < 0.0001). This association was observed in patients with solitary bone metastasis (HR, 0.38; 95% CI, 0.26–0.55; *p* < 0.0001) and in those with multiple bone metastases (HR, 0.38; 95% CI, 0.29–0.51; *p* < 0.0001).

**Figure 3:**
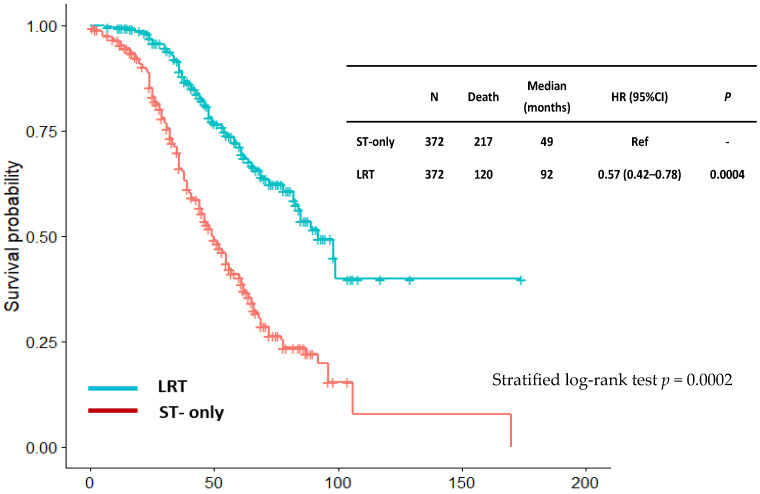
Overall survival comparison between systemic therapy (ST) and locoregional therapy (LRT) groups in patients’ propensity score-matched.

Propensity score matching is used to reduce the bias due to confounding variables that might affect the LRT estimation in this registry study. Comparison of OS between propensity score-matched ST and LRT groups further confirmed better survival for the LRT group (HR: 0.57, 95% Cl: 0.42–0.78) (Figure 3).

In univariate analysis, LRT was associated with significantly improved OS (HR 0.35; 95% CI 0.29–0.45; *p* < 0.0001). Poorer OS was observed in patients older than 52 years (HR 1.30; 95% CI 1.05–1.62; *p* = 0.02), in those with LP (HR 1.92; 95% CI 1.48–2.49; *p* < 0.0001), SP (HR 5.89; 95% CI 4.38–7.93; *p* < 0.0001), and a higher number of metastasis (HR 1.59; 95% CI 1.26–1.99; *p* < 0.0001), while ER/PR(+) was associated with improved OS (HR 0.74; 95% CI 0.56–0.98; *p* = 0.04). In a multivariate Cox proportional model, after adjustment for the baseline and clinical characteristics, LRT (HR 0.49; 95% CI 0.38–0.63; *p* < 0.0001), age older than 52 years (HR 1.29; 95% CI 1.02–1.61; *p* = 0.03), SP (HR 4.84; 95% CI 3.55–6.63; *p* < 0.0001) and ER/PR (+) (HR 0.62; 95% CI 0.46–0.84; *p* = 0.002) remained independent predictors of OS (Table 3). However, LP and solitary metastasis lost their significance. Other factors such as tumor size, tumor type, histologic grade, chemotherapy, and bisphosphonate treatment were not independent predictors of OS (*p* > 0.05).

## 4. Discussion

Distant metastasis is the most important factor shortening survival in BC patients. The St. Gallen International Consensus and ESMO guidelines have increasingly supported curative-intent approaches in carefully selected cases of oligometastatic BC [20,21,22,23]. In 2021, at the St. Gallen International Consensus, patients with low-volume or oligometastatic disease who respond to ST were considered candidates for multimodal treatment with curative intent. This trend continued in subsequent years, with 85–86% of experts supporting the addition of LRT in isolated bone metastases, particularly in HER2-positive or HR-positive patients [20,21]. These evolving recommendations reflect a growing international consensus toward expanding the role of potentially curative strategies in selected dnMBC patients.

Bone is the most frequent site of distant metastasis in BC, and patients with bone-only metastases generally have a more favorable prognosis than those with visceral or brain involvement [24]. Although Marie et al. retrospectively reviewed 242 bone-only MBC patients, stratifying them by age (<50 vs ≥50 years), and found no significant differences in 5-year OS (44.9% vs 39.2%; *p* = 0.21) or 5-year progression-free survival (both 22.7%; *p* = 0.55) [25]. The MF07-01 randomized trial by Soran et al. demonstrated a 29% reduction in the hazard of death (HoD) in the LRT group (HR: 0.71; 95% CI: 0.59–0.86; *p* = 0.0003), particularly in HR-positive, bone-only patients under 55 years [14,26]. Patients with solitary bone-only metastases had 45% less HoD (HR 0.55; 95% CI 0.36–086; *p* = 0.009) at the 10-year follow-up [26]. In addition, a prospective registry showed a 60% reduction in mortality risk in the LRT group [2]. The current BOMETIN registry study substantially expands upon this with a larger cohort, longer follow-up, and consistent findings, reinforcing the survival benefit of LRT in dnBOMBC. In this current analysis, LRT was associated with a 64% reduction in HoD (HR 0.36; 95% CI 0.29–0.45; *p* < 0.0001). In multivariable Cox regression analysis, age older than 52 years was identified as an independent predictor of poorer OS (HR 1.29, 95% CI 1.02–1.61; *p* = 0.03). This large-scale, prospective, multi-national, and multi-institutional registry study provides strong real-world evidence supporting the use of LRT in dnBOMBC. Among 744 patients treated between 2014 and 2022, those receiving LRT had significantly lower mortality (32% vs. 58%, *p* < 0.001), fewer LP (9% vs. 20%, *p* = 0.0001) and SP (41% vs. 66%, *p* < 0.001). This benefit remained consistent across patients with solitary (HR = 0.38; 95% CI: 0.26–0.55) and multiple bone metastases (HR = 0.38; 95% CI: 0.29–0.51), suggesting independence from disease burden. Notably, outcomes did not differ based on the timing of surgery in relation to ST.

Metastatic BC represents a heterogeneous disease spectrum. Our subgroup analysis revealed better outcomes in ER/PR-positive patients (HR = 0.62; *p* = 0.002), while SP was the most significant negative prognostic factor (HR = 4.84; *p* < 0.0001). Similarly, Lane et al. reported that surgery was independently associated with improved OS in ER-positive patients [27]. Several retrospective and prospective studies have evaluated the extent of metastatic burden. Lopez-Tarruella et al. found improved OS with LRT in a large cohort of dnMBC patients, particularly in those with bone or oligometastatic disease [28]. Wang et al. demonstrated that LRT improved survival even in patients with multiple bone lesions and even some limited visceral metastases [29]. In our study, patients with solitary bone metastasis were more likely to receive LRT (50% vs. 24%; *p* < 0.001). However, survival benefit from LRT persisted in both solitary- and multiple-metastasis subgroups.

While many observational studies with a large sample size support the survival benefit of surgery, some RCTs have not demonstrated a significant OS advantage in unselected dnMBC populations [9,10,30,31]. These RCTs had some limitations, such as patients not receiving standard care of ST agents, early termination of the study with a very limited patient enrollment, heterogeneous patient characteristics, or suboptimal surgical techniques. A pooled meta-analysis of 7 RCTs including 1018 patients found no OS benefit (HR: 0.87; 95% CI: 0.68–1.11; *p* = 0.265), though LP-free survival was significantly improved (HR: 0.27; 95% CI: 0.19–0.38; *p* < 0.001) [32]. Although elimination of dissemination of disease from the primary tumor, control of metastatic disease prompted by removal of the primary tumor, and eliminating the possibility of the primary tumor producing factors that promote the growth of distant disease are proposed reasons that might improve survival, biological hypotheses suggest that surgical removal of the primary tumor may disrupt dormancy signals or promote circulating tumor cell dissemination [33,34,35]. The Cochrane review by Tosello et al. concluded that LRT improved local control (HR: 0.22; 95% CI: 0.08–0.57), but was associated with worse distant progression (HR: 1.42; 95% CI: 1.08–1.86), possibly due to delays or interruptions in ST [36]. The ECOG-ACRIN trial reported improved local control with LRT and no benefit in OS, but was criticized for inadequate margins and a high percentage of patients with advanced local disease [30]. Similarly, Reinhorn et al.’s pooled analysis of four RCTs showed no OS benefit regardless of subtype or metastatic pattern [37]. By contrast, in our study with anthracycline-taxane-based chemotherapy, anti-HER2 therapy, endocrine therapy (aromatase inhibitors or tamoxifen/ovarian suppression), and bone-modifying agents, LRT reduced mortality from 58% to 32% (*p* < 0.001), extended median OS from 49 months to 92 months (ST+LRT) and 99 months (LRT+ST), decreased LP from 20% to 9% (*p* = 0.0001), and decreased SP from 66% to 41% (*p* < 0.001), underscoring the value of optimal patient selection and integration of LRT with modern ST in this population.

To better define candidates for LRT, predictive models and nomograms have been proposed. Kommalapati et al. analyzed 67,978 dnMBC patients to create a prognostic scoring system, and they found that the patients who received LRT had significantly improved median OS (45 months) as compared to those who did not (24 months) (*p* < 0.0001) [38]. Although it awaits validation, a SEER-based model has shown promise for a new prognostic staging system for de novo MBC [39]. A study comparing MD Anderson and Jeanny nomograms in 72 dnBOMBC patients found better predictive performance in surgically treated patients [40]. Goktepe et al. published a study of a combination of data from a phase 3 randomized trial and a prospective multi-institutional registry trial and in this study, LRT significantly improved OS in patients with Her 2-positive, HR-positive and low grade tumor and less than 5 cm tumors (solitary: HR, 0.375, 95% CI 0.259–0.543, *p* < 0.001; multiple: HR 0.435, 95% CI 0.334–0.615, *p* <0.001) [41]. Conversely, in the group of patients who had high grades, T4, and triple-negative tumors, there was no significant benefit of OS from LRT. These results underline the need for individualized, surgery-specific prognostic tools.

The strengths of our study include its large prospective registry design, long follow-up (median 48 months), and multi-institutional collaboration. LRT remained an independent predictor of improved OS in both surgical subgroups in univariate and multivariable Cox regression models. Although this study has a greater sample size, there are some limitations; patients in the LRT group were younger (median age 50 vs. 55 years; *p* = 0.0001), had more solitary bone lesions (50% vs. 24%; *p* < 0.001), and more frequently received chemotherapy (95% vs. 87%; *p* = 0.0005), suggesting possible selection bias. Limitations also include a lack of quality-of-life data, detailed radiotherapy fields, and surgical techniques. Accrual (2014–2022) overlaps with the use of newer ST agents such as CDK4/6 inhibitors, newer anti-HER2 agents, and immunotherapy; because agent-level therapies were not uniformly collected, confounding by treatment era cannot be excluded. On the other hand, to reduce the bias due to confounding variables, propensity score matching is used. Comparison of OS between propensity score-matched ST and LRT groups further confirmed better survival for the LRT group (HR: 0.57, 95% Cl: 0.42–0.78) (Figure 3).

In conclusion, this large prospective registry study focused exclusively on dnBOMBC provides the most recent high-level real-world evidence that LRT was associated with longer survival and better disease control. Future prospective trials should further refine patient selection and incorporate quality-of-life outcomes to inform personalized, multidisciplinary treatment strategies.

## Figures and Tables

**Table 1:** Baseline characteristics between systemic therapy (ST) and locoregional therapy (LRT) groups.

	ST Group *n* = 372 (%)	LRT Group *n* = 372 (%)	*p* Value
Median follow-up (25%, 75%)	39 (26, 58)	58 (38, 74)	<0.001
Median age (25%, 75%)	55 (44, 66)	50 (42, 60)	0.0001
Median Body mass index (25%, 75%)	28 (25, 31)	27 (25, 31)	0.39
Locoregional progression	76 (20)	32 (9)	0.0001
Systemic progression	244 (66)	152 (41)	<0.001
Mortality rate	217 (58)	120 (32)	<0.001
Number of metastases			<0.001
Solitary	89 (24)	185 (50)	
Multiple (>1)	283 (76)	187 (50)	
Tumor size			0.009
T1	66 (18)	60 (16)	
T2	246 (66)	219 (59)	
T3	47 (13)	84 (23)	
T4	11 (3)	9 (2)	
Tumor type			0.0005
Invasive Ductal Carcinoma	286 (77)	313 (84)	
Invasive Lobular Carcinoma	58 (16)	25 (7)	
Others	27 (7)	34 (9)	
Histologic grade			0.02
1	52 (14)	32 (9)	
2	162 (45)	188 (51)	
3	129 (36)	140 (38)	
Missing	16 (4)	8 (2)	
Her2 status			0.36
Negative	279 (75)	268 (72)	
Positive	93 (25)	104 (28)	
Estrogen/Progesterone receptors			0.04
Negative	41 (11)	60 (16)	
Positive	331 (89)	312 (84)	
Triple-Negative			0.28
No	353 (95)	346 (93)	
Yes	19 (5)	26 (7)	
Hormonotherapy	317 (85)	317 (85)	0.99
Chemotherapy	322 (87)	353 (95)	0.0005
Bisphosphonate treatment	260 (70)	231 (62)	0.02
Ovarian suppression	71 (19)	88 (24)	0.13
Intervention to metastasis	188 (51)	198 (53)	0.46

**Table 2:** Baseline characteristics among systemic therapy (ST) and locoregional therapy (LRT); subgroups defined as LRT+ST and ST+LRT groups.

	ST Group *n* = 372 (%)	LRT+ST Group *n* = 221 (%)	ST+LRT Group *n* = 151 (%)	*p* Value
Median follow-up (25%, 75%)	39 (26, 58)	59 (40, 74)	53 (38, 73)	<0.001
Median age (25%, 75%)	55 (44, 66)	50 (43, 59)	49 (40, 60)	0.0001
Median BMI (25%, 75%)	28 (25, 31)	27 (24, 31)	27 (25, 31)	0.69
Locoregional progression	76 (20)	23 (10)	9 (6)	0.0001
Systemic progression	244 (66)	87 (39)	65 (43)	<0.001
Mortality rate	217 (58)	76 (34)	44 (29)	<0.001
Number of metastases				<0.001
Solitary	89 (24)	125 (57)	60 (40)	
Multiple (>1)	283 (76)	96 (43)	91 (60)	
Tumor size				0.008
T1	66 (18)	42 (19)	18 (12)	
T2	246 (66)	132 (60)	87 (58)	
T3	47 (13)	44 (20)	40 (26)	
T4	11 (3)	3 (1)	6 (4)	
Tumor type				0.002
Invasive Ductal Carcinoma	286 (77)	186 (84)	127 (84)	
Invasive Lobular Carcinoma	58 (16)	12 (5)	13 (9)	
Others	27 (7)	23 (10)	11 (7)	
Histological grade				0.036
1	52 (14)	18 (8)	14 (10)	
2	162 (45)	111 (50)	77 (52)	
3	129 (36)	84 (38)	56 (38)	
Missing	16 (4)	8 (4)	0 (0)	
Her2 status				0.40
Negative	279 (75)	155 (70)	113 (75)	
Positive	93 (25)	66 (30)	38 (25)	
Estrogen/Progesterone receptors				0.05
Negative	41 (11)	40 (18)	20 (13)	
Positive	331 (89)	181 (82)	131 (87)	
Triple-negative				0.07
No	353 (95)	201 (91)	145 (96)	
Yes	19 (5)	20 (9)	6 (4)	
Hormonotherapy	317 (85)	182 (82)	135 (89)	0.17
Chemotherapy	322 (87)	205 (93)	148 (98)	0.0002
Bisphosphonate treatment	260 (70)	149 (67)	82 (54)	0.003
Ovarian suppression	71 (19)	49 (22)	39 (26)	0.22
Intervention to metastasis	188 (51)	99 (45)	99 (66)	0.0003

**Table 3:** Univariate and Multivariable Cox models for overall survival.

Parameter	HR (95%CI)	*p*	HR_adj_ (95%CI)	*p*_adj_
Locoregional therapy	0.35 (0.29–0.45)	<0.0001	0.49 (0.38–0.63)	<0.0001
Age > 52 (median age)	1.30 (1.05–1.62)	0.02	1.29 (1.02–1.61)	0.03
Locoregional progression	1.92 (1.48–2.49)	<0.0001	1.18 (0.89–1.55)	0.25
Systemic progression	5.89 (4.38–7.93)	<0.0001	4.84 (3.55–6.63)	<0.0001
Number of metastases	1.59 (1.26–1.99)	<0.0001	1.21 (0.96–1.54)	0.11
Primary tumor size	1.09 (0.93–1.28)	0.28	1.06 (0.89–1.27)	0.51
Tumor type	1.02 (0.85–1.22)	0.85	0.93 (0.77–1.13)	0.48
Histologic grade	1.01 (0.87–1.18)	0.88	0.97 (0.83–1.14)	0.71
ER/PR (+)	0.74 (0.56–0.98)	0.04	0.62 (0.46–0.84)	0.002
Chemotherapy received	0.83 (0.56–1.22)	0.33	1.30 (0.81–2.09)	0.27
Bisphosphonate treatment received	1.08 (0.85–1.37)	0.54	1.00 (0.77–1.30)	0.98

## Data Availability

The data presented in this study are available upon reasonable request from the corresponding author. The data are not publicly available due to privacy and ethical restrictions.

## Abbreviations

ST	Systemic therapy
dnBOMBC	De novo bone-only metastatic breast cancer
dnMBC	De novo metastatic breast cancer
HR	Hormone receptor
HER2	Human epidermal growth factor receptor 2
ER	Estrogen receptor
PR	Progesterone receptor
LP	locoregional progression
LRT	locoregional treatment
SP	Systemic progression
CDK	cyclin-dependent kinase
RCT	Randomized controlled trial
HR	Hazard ratio
CI	Confidence interval
OS	Overall survival
NCCN	National Comprehensive Cancer Network
